# Phosphatidylserine Exposure by *Toxoplasma gondii* Is Fundamental to Balance the Immune Response Granting Survival of the Parasite and of the Host

**DOI:** 10.1371/journal.pone.0027867

**Published:** 2011-11-29

**Authors:** Thiago Alves Teixeira dos Santos, Juliana de Araújo Portes, João Claudio Damasceno-Sá, Lucio Ayres Caldas, Wanderley de Souza, Renato Augusto DaMatta, Sergio Henrique Seabra

**Affiliations:** 1 Laboratório de Tecnologia em Bioquímica e Microscopia (LTBM), Colegiado de Ciências Biológicas e da Saúde, Centro Universitário Estadual da Zona Oeste (UEZO), Rio de Janeiro, Brazil; 2 Laboratório de Biologia Celular e Tecidual, Centro de Biociências e Biotecnologia, Universidade Estadual do Norte Fluminense Darcy Ribeiro (UENF), Campos dos Goytacazes, Brazil; 3 Laboratório de Ultraestrutura Celular Hertha Meyer, Instituto de Biofísica Carlos Chagas Filho, Universidade Federal do Rio de Janeiro (UFRJ), Rio de Janeiro, Brazil; AC Camargo Cancer Hospital, Brazil

## Abstract

Phosphatidylserine (PS) exposure on the cell surface indicates apoptosis, but has also been related to evasion mechanisms of parasites, a concept known as apoptotic mimicry. *Toxoplasma gondii* mimics apoptotic cells by exposing PS, inducing secretion of TGF-beta1 by infected activated macrophages leading to degradation of inducible nitric oxide (NO) synthase, NO production inhibition and consequently persisting in these cells. Here PS^+^ and PS^−^ subpopulation of tachyzoites were separated and the entrance mechanism, growth and NO inhibition in murine macrophages, and mice survival and pathology were analyzed. Infection index in resident macrophages was similar for both PS subpopulations but lower when compared to the total *T. gondii* population. Growth in resident macrophages was higher for the total *T. gondii* population, intermediate for the PS^+^ and lower for the PS^−^ subpopulation. Production of NO by activated macrophages was inhibited after infection with the PS^+^ subpopulation and the total populations of tachyzoites. However, the PS^−^ subpopulation was not able to inhibit NO production. PS^+^ subpopulation invaded macrophages by active penetration as indicated by tight-fitting vacuoles, but the PS^−^ subpopulation entered macrophages by phagocytosis as suggested by loose-fitting vacuoles containing these tachyzoites. The entrance mechanism of both subpopulations was confirmed in a non-professional phagocytic cell line where only the PS^+^ tachyzoites were found inside these cells in tight-fitting vacuoles. Both subpopulations of *T. gondii* killed mice faster than the total population. Clear signs of inflammation and no tachyzoites were seen in the peritoneal cavity of mice infected with the PS^−^ subpopulation. Moreover, mice infected with the PS^+^ subpopulation had no sign of inflammation and the parasite burden was intense. These results show that PS^+^ and PS^−^ subpopulations of *T. gondii* are necessary for a successful toxoplasma infection indicating that both subpopulations are required to maintain the balance between inflammation and parasite growth.

## Introduction

Toxoplasmosis is caused by *Toxoplasma gondii*, an obligate intracellular protozoan [1]. This parasite has a worldwide distribution, in a broad host range, and is considered one of the most successful on earth [1], [2]. Although up to one third of the world human population is infected with *T. gondii* [3], most infections are asymptomatic, but severe clinical manifestations can arise in immunocompromised individuals [1].

Although NO production by activated macrophages controls *T. gondii* proliferation [4]–[7], the parasite regulates NO production and can persist in activated macrophages [4], [5], [7]–[9]. Our group has showed that approximately 50% of the total population of *T. gondii* exposes phosphatidylserine (PS) on their outer leaflet of the plasma membrane, this mechanism is involved in the inhibition of NO production of infected activated macrophages [4]. Inhibition of NO allows *T. gondii* to persist in infected macrophages [4], [5], [7]–[9]. A reduced expression of inducible NO synthase [4], [5], [7], [8] and disappearance of nuclear factor kappa B (NF-κB) from the nucleus of activated *T. gondii* infected macrophages occurs in a Transforming Growth Factor-beta1 (TGF-b1) dependent way [4]. However, the molecular mechanism that controls these evasion processes is not well known.

PS is a phospholipid present at the plasma membrane, which is a major ligand involved in the uptake of apoptotic cells [10]. Phagocytosis of apoptotic cells by macrophages induces a noninflammatory response based on the exposure of PS [11] that leads to TGF-b1 secretion [11], [12]. Due to this property, PS has been related to the evasion mechanism of *Leishmania amazonensis* from macrophages, a concept known as “apoptotic mimicry” [13], [14]. It was demonstrated that this protozoan exposes PS and this is involved in the internalization process, causing alternative activation of macrophage through the induction of TGF-b1 secretion, interleukin (IL)-10 synthesis, and inhibition of NO production [15], [16]. Similarly, trypomastigotes of *Trypanosoma cruzi* exposes PS reducing iNOS expression after infection of activated macrophages [17]. Apoptotic mimicry has also been implicated in the entrance of the vaccinia virus into host cells [18].

The mechanism of *T. gondii* invasion involves the formation of a moving junction between the parasite and the host cell plasma membrane. The plasma membrane invaginates concomitantly with the formation of the parasitophorous vacuole. This process is known as active invasion [19]. However, the entrance of tachyzoites can also occur by an internalization pathway that involves the host cell [19], [20], as recently indicated in a study using dynasore [21], an inhibitor of the endocytic pathway [22].

Here we showed the immunopathological mechanisms behind the interaction of isolated PS^+^ and PS^−^ subpopulation of *T. gondii* with host cells. Morphological analyses of their interactions with dynasore showed that PS^+^ parasites invaded macrophages by active penetration, while PS^−^ parasites entered by phagocytosis. Only the PS^+^ subpopulation of *T. gondii* was able to inhibit NO of activated macrophages. A non-professional phagocytic cell line was actively invaded by the PS^+^ subpopulation, but no tachyzoites were internalized in this cell line when the PS^−^ subpopulation was used. Furthermore, infected mice with the separated subpopulations were killed faster than the ones infected with the total population. High levels of inflammation were found in mice infected with the PS^−^ subpopulation, and increased parasite burden was present in mice infected with the PS^+^ subpopulation, explaining the low survival of mice infected with both subpopulations. Collectively, these results indicate that the escape mechanism of *T. gondii* is dependent on the exposure of PS and suggests that both subpopulations of *T. gondii* are necessary for a successful infection and survival.

## Results

### 1. The isolation method to obtain PS^+^ and PS^−^ subpopulations was efficient

**Figure 1:**
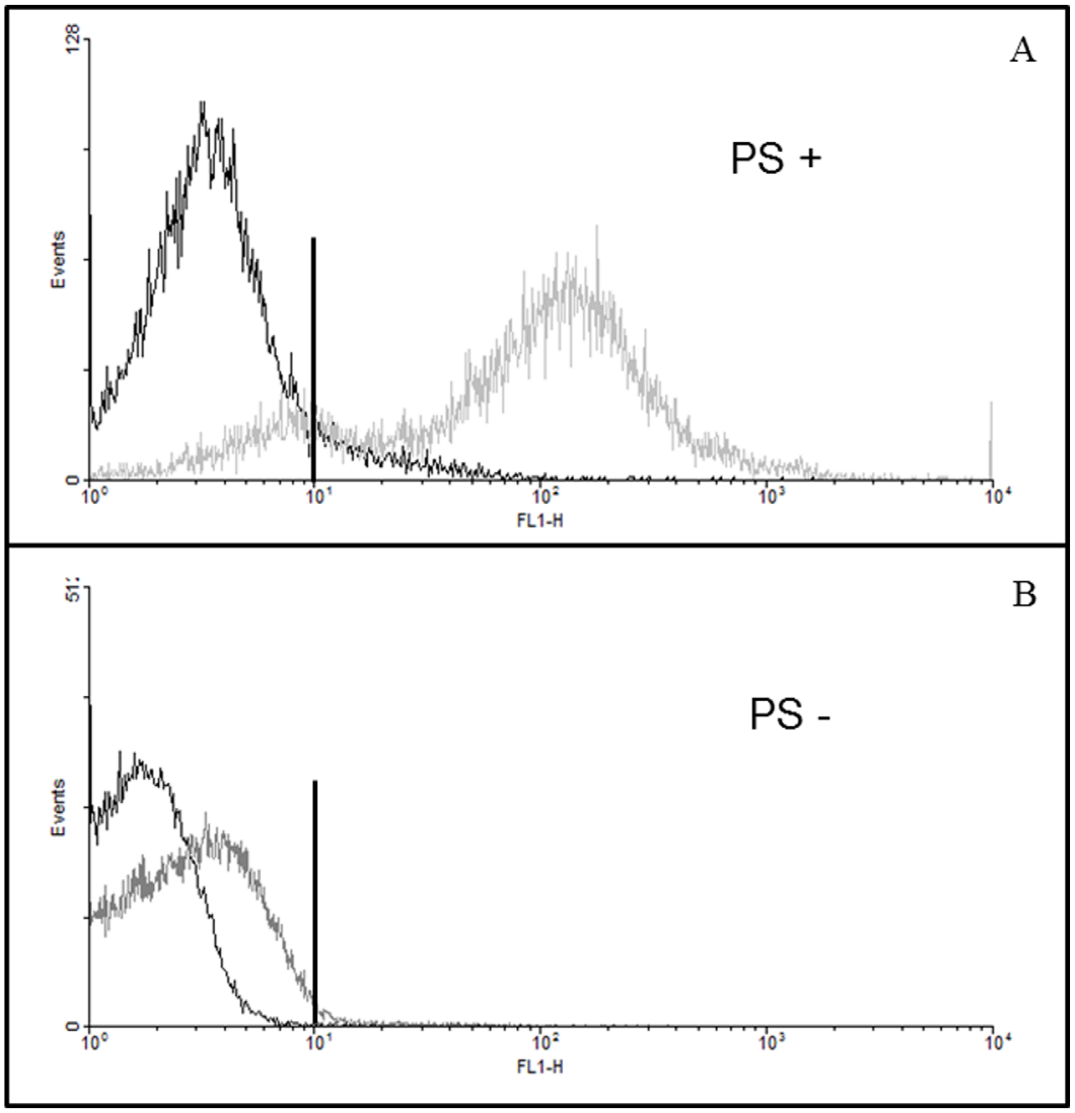
Phosphatidylserine (PS)^+^ and PS^−^ subpopulations of *Toxoplasma gondii* were magnetically separated and analyzed by flow cytometry after annexin-V staining. (A) PS^+^ subpopulation of *T. gondii*. (B) PS^−^ subpopulation of *T. gondii*. The black lines represent control parasites without Annexin – V and the gray lines refer to the isolated subpopulations. Results from one representative experiment out of five.

The isolation method of the PS^+^ and PS^−^ tachyzoite subpopulation was assayed by flow cytometer. This analysis showed a clear shift to the right of the histogram indicating that the isolated PS^+^ subpopulation was exposing high levels of PS (Figure 1A). A 96% purity was obtained for the PS^+^ subpopulation. In addition, the isolated PS^−^ subpopulation did not stain with annexin V (Figure 1B); a 98% purity was obtained.

### 2. The PS^−^ subpopulation of tachyzoite entered macrophages through phagocytosis and only the PS^+^ subpopulation inhibited NO of activated macrophages

**Figure 2:**
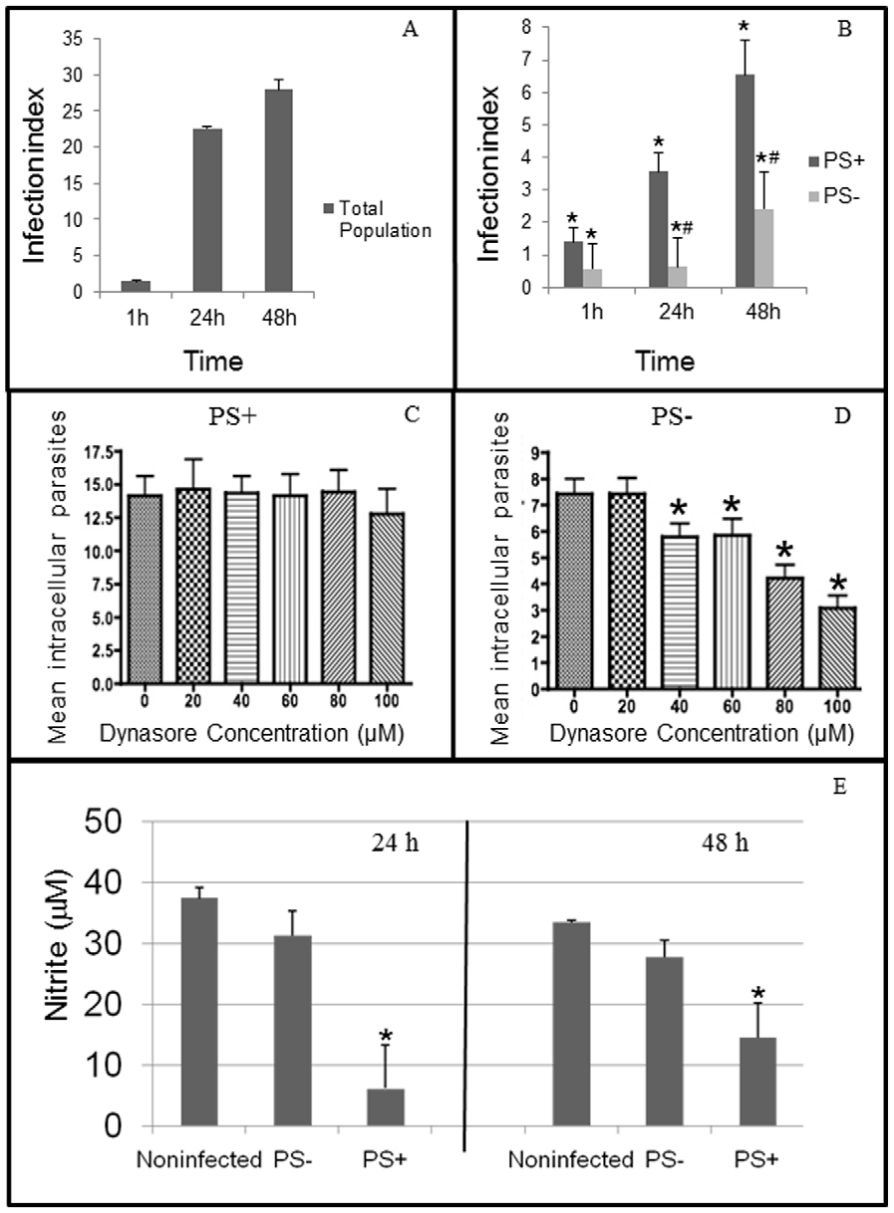
Interaction of *Toxoplasma gondii* total population, PS^+^ or PS^−^ subpopulations of tachyzoites with resident (A, B, C, D) or activated (E) macrophages. (A) Infection index of resident macrophage after interaction with total population. (B) Infection index of resident macrophage after interaction with PS^+^ or PS^−^ *T. gondii* subpopulations. *Significant difference from total population (*p*≤0.05) by variance analysis (ANOVA). #Significant difference from the PS^+^ subpopulation (*p*≤0.05) by variance analysis (ANOVA). (C–D) Infection index of resident macrophage that interacted with PS^+^ (C) or PS^−^ (D) *T. gondii* subpopulation in the presence of dynasore. *Significant difference from the untreated samples (*p*≤0.05) by variance analysis (ANOVA). (E) Nitric oxide production of noninfected activated macrophages or after interaction with PS^+^ or PS^−^ subpopulations of *T. gondii*. *Significant difference from the noninfected values (*p*≤0.05) by the Student *t* test. Results are from at least three independent experiments.

Both isolated PS subpopulations were less capable of infecting resident macrophages after 1 h when compared to the total *T. gondii* population (Figure 2A and 2B). However, the PS^+^ subpopulation was better able to grow in resident macrophages after 24 and 48 h when compared to the PS^−^ subpopulation (Figure 2B). To further understand the entrance mechanism of both PS subpopulations in resident macrophages, dynasore was used before and during the interaction. The entrance capacity of the PS^+^ subpopulation did not vary with increasing concentrations of dynasore (Figure 2C); however, the entrance capacity of the PS^−^ subpopulation lowered with higher dynasore concentrations (Figure 2D). Moreover, only the PS^+^ subpopulation was able to inhibit NO production after 24 and 48 h of infection of activated macrophages (Figure 2E). As demonstrated before, the total population inhibited NO production like the PS^+^ subpopulation (not shown). Furthermore, the amount of nitrite measured in the supernatant of activated macrophages that interacted with the PS^−^ subpopulation was statistically equal to the amount produced by noninfected activated macrophages (Figure 2E).

### 3. PS^−^ tachyzoites were in loose-fitting vacuoles while the PS^+^ were in tight-fitting vacuoles

**Figure 3:**
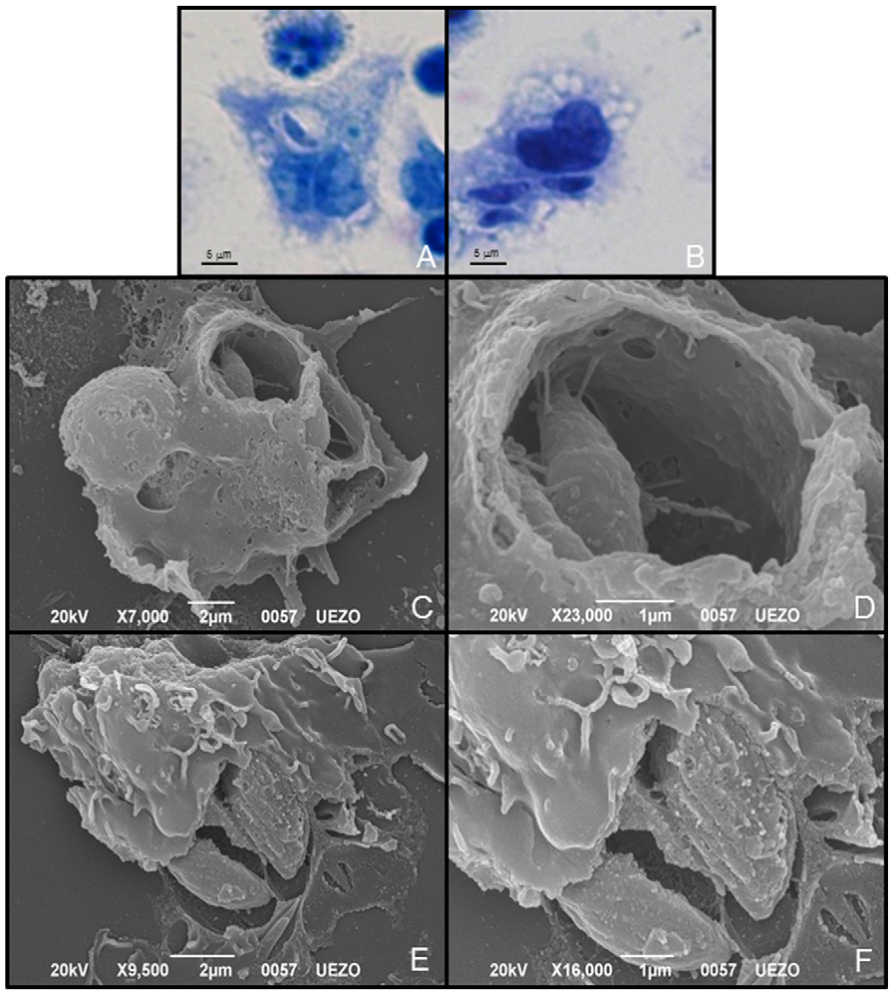
Light and scanning electron microscopy of macrophage vacuoles with phosphatidylserine (PS)^+^ and PS^−^ tachyzoites of *Toxoplasma gondii*. (A), (C) and (E) Macrophage vacuoles after interaction with PS^−^ subpopulation of *T. gondii*. Note the loose-fitting vacuole around the parasite caused by phagocytosis entrance. (B), (D) and (F) Macrophage vacuoles after interaction with PS^+^ subpopulation of *T. gondii*. Note the tight-fitting vacuoles around the parasite, indicating active penetration. Results are from three independent experiments.

**Figure 4:**
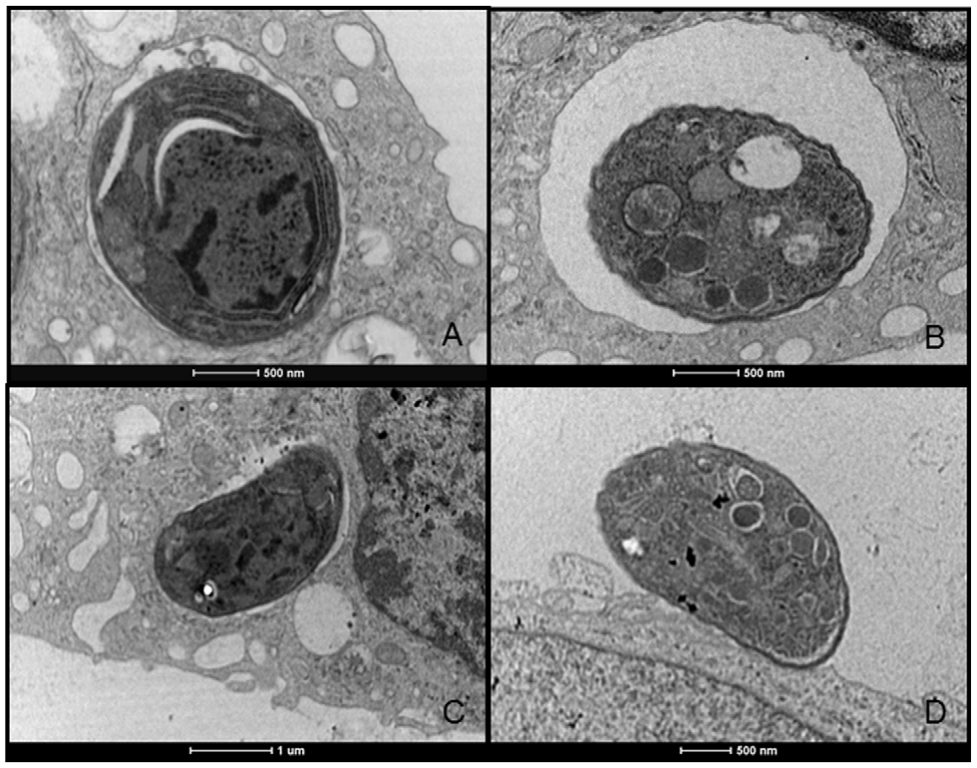
Transmission electron microscopy of vacuoles containing *Toxoplasma gondii* after interactions with macrophages or the non-phagocytic cell line LLC-MK2. (A) Interaction of macrophages with PS^−^ subpopulation of *T. gondii*. (B) Interaction of macrophages with PS^+^ subpopulation of *T. gondii*. (C) Interaction of LLC-MK2 with PS^−^ subpopulation of *T. gondii*. (D) Interaction of LLC-MK2 with PS^+^ subpopulation of *T. gondii*. Note tight-fitting vacuoles after interaction of host cells with PS^+^ tachyzoites; PS^−^ tachyzoites were not able to enter LLC-MK2 cells and were found in loose-fitting vacuoles after interaction with macrophages. Results are from two independent experiments.

Loose-fitting vacuoles observed after the interaction of macrophages with the PS^−^ subpopulation (Figures 3A, 3C, 3E and 4A) are regarded as an indicator of phagocytosis as the entrance mechanism of tachyzoites [19]. Interaction of macrophages with the PS^+^ subpopulation resulted in tight-fitting vacuoles (Figures 3B, 3D, 3F and 4B), indicating that active penetration [19] was the main entrance mechanism in macrophages for this subpopulation. Tight and loose-fitting vacuoles were counted by light microscopy and interactions with the PS^−^ subpopulations resulted in about 76% of loose-fitting vacuoles and the PS^+^ subpopulation in 78% of tight-fitting vacuoles.

Analysis by transmission electron microscopy of the interaction between the non-professional phagocytic LLC-MK2 and isolated subpopulations of *T. gondii* showed that the PS^−^ parasites could not infect the host cells (Figure 4C). However, the PS^+^ parasites were able to invade this cell lineage (Figure 4D) by active penetration as judged by the tight-fitting vacuole.

### 4. Both PS subpopulations were able to kill mice faster than the total population

**Figure 5:**
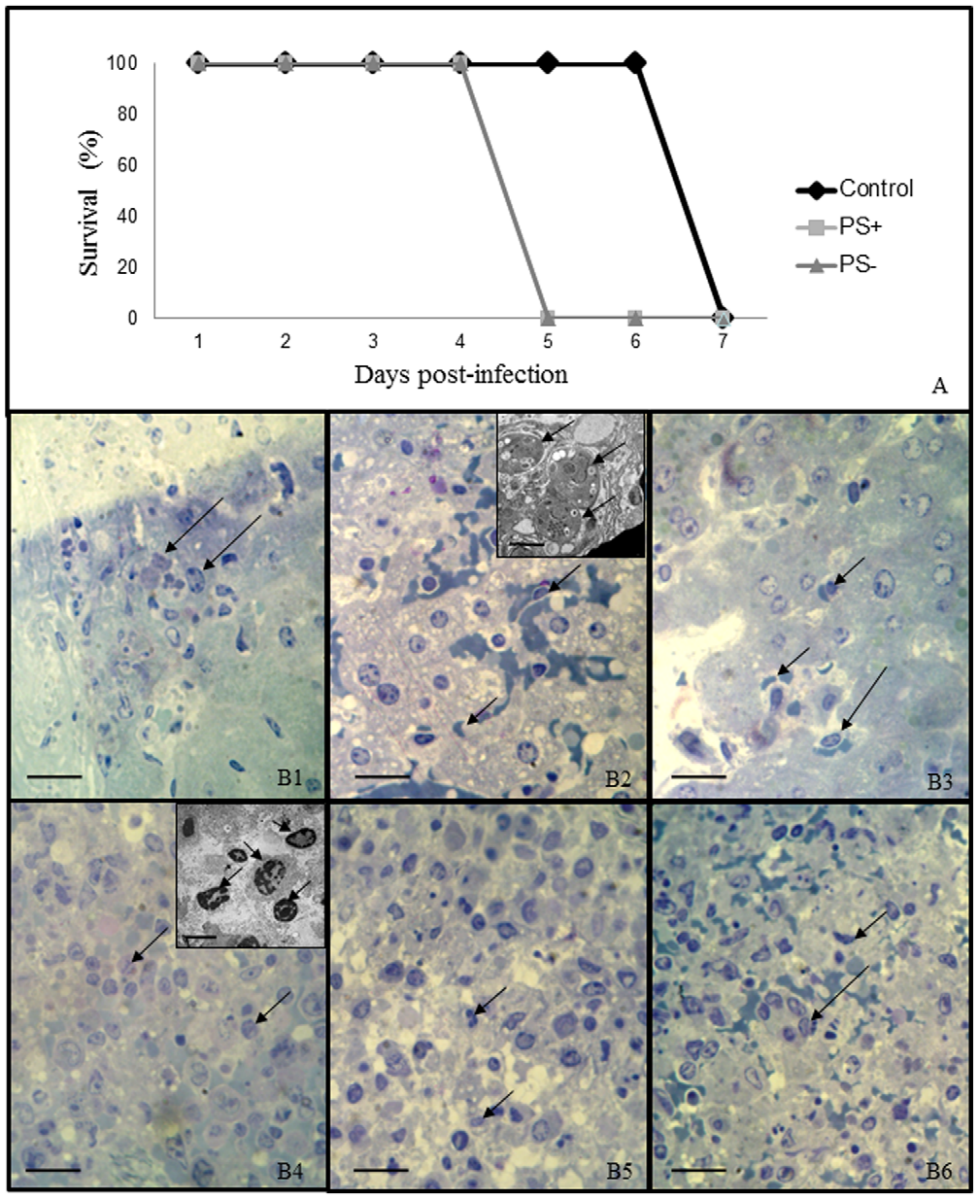
Analysis of *Toxoplasma gondii* infection *in vivo*. C57/BL6 mice were infected with PS^+^, PS^−^ subpopulations or the total population of *T. gondii*. (A) Survival curve of mice after the infection with *T. gondii*. Kaplan Meier analysis *p* = 0.0273. (B) Light microscopy of spleen and liver tissue of C57/BL6 mice after interaction with PS^+^ or PS^−^ subpopulation of *T. gondii*. Bars = 100 µm. (B1–3) Spleen images after interaction with the PS^−^ (B1), PS^+^ (B2) or the total population of *T. gondii* (B3). Note the presence of inflammatory cells in B1 (arrows) and the presence of parasites in B2 and B3 (arrows). (B4–6) Liver images after interaction with PS^−^ (B4), PS^+^ (B5) or the total population of *T. gondii* (B6). Note similar results obtained for the spleen tissue. Inset – Transmission electron microscopy: B2, Bar = 2 µm; B4, Bar = 16 µm. Results are from two independent experiments with 6 animals per group.

Mice infected with PS^+^ or PS^−^ subpopulations of *T. gondii* had a mean survival time of 5 days, while mice infected with the total population of parasites had a mean survival time of 7 days (Figure 5A). Immediately following the death of the mice, spleen and liver were collected for pathological analysis. Leukocytes of the inflammatory infiltrate and parasites were counted and cell numbers in tissues of mice infected with the PS subpopulations were compared to the numbers found in tissue of mice infected with the total population. Macrophages and lymphocytes were the only leukocyte types seen and the former the only one with significant differences between the infections. Spleens infected with the PS+ subpopulation presented a significant reduction (2.75 fold) in the number of macrophages and a significant increase (2.20 fold) of tachyzoites (5B2 and 5B3). The infection with the PS^−^ subpopulation resulted in a significant increase of macrophages (1.91 fold) in relation to the infection with the total population (5B1 and 5B3). In livers, the infection with the PS+ subpopulation decreased significantly the number of macrophages (3.00 fold) and increased significantly the number of tachyzoites (2.67 fold) (5B5 and 5B6). The infection with the PS− subpopulation increased significantly the number of macrophage (1.91 fold) and decreased significantly the number of tachyzoites (2.00 fold) (5 B4 and 5B6).

## Discussion

*Toxoplasma gondii* is an obligate intracellular parasitic protozoan that causes toxoplasmosis. The success of the infection by this parasite depends on the evasion mechanisms against the host's immune response. However, the parasite population must be controlled to avoid host death, ensuring parasite survival and subsequent passage to the next generation of the host. The balance between parasite evasion mechanism and host control of the parasite is not understood. In studies with *L. amazonensis* it has been reported that exposure of PS on the surface of the parasites is one of the main mechanisms of inhibition of the immune response [15], [16]; leading to the concept of “apoptotic mimicry” [13], [14]. When a cell initiates the apoptotic process, PS on the outer extracellular leaflet of the plasma membrane signals the inhibition of the inflammatory response of macrophages [11], [23]. In the quest to better understand the behavior of the infection caused by the PS^+^ and PS^−^ subpopulations of *T. gondii*, interactions were performed *in vitro* and *in vivo* after the isolation of these subpopulations by magnetic separation. Flow cytometry assured the efficacy of PS subpopulations isolation.

A reduced infection index in resident macrophages was observed when both PS subpopulations were used to infect these cells compared to the total *T. gondii* population. To better understand this result, analyses of the size of the vacuoles containing tachyzoites were performed by light, scanning and transmission electron microscopy, through which we could observe that PS^−^ parasites were inside loose-fitting vacuoles and PS^+^ parasites inside tight-fitting vacuoles. In addition, interaction between isolated subpopulations and the non-professional phagocytic cell line, LLC-MK2, showed that only PS^+^ parasites were internalized. It has been demonstrated that tachyzoites that infect the host cells by active penetration end up in tight-fitting vacuole, whereas the ones that enter through phagocytosis are found in loose-fitting vacuole [19]. Thus, our results indicate that PS^−^ subpopulation enter macrophages through a phagocytic mechanism. On the other hand, the PS^+^ subpopulation was found in macrophages and in the LLC-MK2 cell line in tight-fitting vacuoles. Because this type of vacuoles is an indicator of active penetration [19], it is reasonable to conclude that only the PS^+^ tachyzoite were able to use this entrance mechanism to invade host cells. Furthermore, this difference in entrance mechanism between both PS subpopulations and the fact that the LLC-MK2 cell line is not a professional phagocyte, explains why PS^−^ tachyzoites were not found inside this cell line.

Dynasore has been described as an inhibitor of the endocytic pathway because it inhibits dynamin [22]. Dynamin has recently been implicated in actin dynamics [24], thus, its use can alter all cell processes that involve this cytoskeleton filament, including phagocytosis. Moreover, phagocytosis also depends on the fusion of endomembrane compartments to the plasma membrane to form the phagosome, a process known as “focal exocytosis” [25]. Because dynamin is necessary for the fusion and scission of vesicles [26], it is sound to infer that dynasore is a potential inhibitor of phagocytosis. The use of dynasore reduced the entrance of the PS^−^ subpopulation, but no alteration was found when the PS^+^ subpopulation was treated. This corroborates with phagocytosis as the internalization mechanism in macrophages by the PS^−^ subpopulation. Furthermore, this compound was not able to inhibit the active penetration mechanism of PS^+^ subpopulation indicating that dynasore only worked at host cell actin filaments.

Another interesting datum was that the infection index in resident macrophages was higher for the total population when compared to both PS subpopulations. This result can be explained by the fact that PS^+^ and PS^−^ subpopulations entered macrophages by active penetration or phagocytosis, respectively. Because the total population was a combination of both subpopulations that enter through two distinct mechanisms, the infection index for the total population was higher compared to each PS subpopulations, which enter by one or the other mechanism. The infection index in resident macrophages was higher for the PS^+^ subpopulation than for PS^−^ parasites. This result indicates that only PS^+^ tachyzoites are capable of developing in resident macrophages. Because the PS^−^ subpopulation enters macrophages through phagocytosis, this may lead to more effective killing mechanisms, suppression of growth and lower infection index.

It has been reported that 50% of the tachyzoite population are found in macrophages in loose-fitting vacuoles [20]. On the other hand, it has also been reported that 80% of the tachyzoite population that enter macrophages are found in tight-fitting vacuoles and 20% in loose-fitting vacuoles [19]. This might be explained by the percentage of the PS^+^ tachyzoite that composes the population. In our model, the PS^+^ subpopulation varies from 50 to 80% of the total population (unpublished results). All together the datum indicates that PS exposure may be important to trigger the active penetration process. Nevertheless, further studies are necessary to better understand the consequences of PS exposure on parasite-host cell interactions.

Quantification of NO production during interaction with isolated tachyzoite subpopulations, as well as with the total population of *T. gondii*, showed that only the total and the PS^+^ subpopulation were able to inhibit the production of this microbicidal agent, whereas PS^−^ subpopulation was not able to inhibit NO production in activated macrophages. These results confirm the hypothesis that only parasites that expose PS can inhibit NO production by macrophages [4]. Thus, only these tachyzoites are able to escape this microbicidal system ensuring the persistence of *T. gondii* in activated macrophages. This is a clear confirmation that apoptotic mimicry is used by *T. gondii* [4], [27].

Infection of mice with both subpopulations and the total population of *T. gondii* showed that both isolated subpopulations caused an early death compared to mice infected with the total population. Light and transmission electron microscopy of liver and spleen of infected mice revealed that mice infected by the PS^+^ subpopulation died through hyper parasitemia, indicating that the immune system of these mice was not able to contain and control the proliferation of this subpopulation. On the other hand, mice infected with the PS^−^ subpopulation died of a high inflammatory process probably generated by their immune systems as a response against this subpopulation of tachyzoites. This is likely to happen because PS^−^ tachyzoites were not capable of inhibiting the antiparasitic activity caused by NO, inducing a higher inflammatory response not balanced by the PS^+^ subpopulation. The examined liver and spleen of mice infected with the total population of *T. gondii* revealed the presence of mild parasitosis and inflammatory infiltrate, suggesting that the higher survival rate of these mice compared with the ones infected with separated PS subpopulations was due to an equilibrium between the host immune response and the proliferation of the *T. gondii*.

In conclusion, *T. gondii* of the RH strain can be divided into two subpopulations with different PS exposure capacities. These subpopulations gain access to macrophages in two distinct manners resulting in completely different final outcomes: survival or death of the parasite. Both subpopulations may be necessary to maintain a balance in the host between parasite death and growth. This balance may be achieved by the different PS subpopulations assuring parasite and host survival resulting in an evident advantage to the RH strain of *T. gondii*.

## Materials and Methods

### 1. Peritoneal macrophages and LLC-MK2

Peritoneal macrophages were obtained by peritoneal washing of Swiss mice with Hank's solution [5]. Macrophages were plated over glass coverslips in 24-well plates. After 1 h of adherence at 37°C, cells were washed with Hank's solution and cultured with DMEM containing 5% FBS at 37°C in a 5% CO_2_ atmosphere. LLC-MK2 (ATCC number CCL-7™) was maintained in 25 cm^2^ cell culture flasks bottles with DMEM containing 10% FBS. Cells were infected with the separated PS subpopulations of tachyzoites (see item 4) and fixed for transmission electron microscopy (item 11).

### 2. Ethics Statement

This study was carried out in strict accordance with the Brazilian Law #11794/08. The animal studies protocol was reviewed and approved by the Committee on the Ethics of Animal Experiments of the Universidade Estadual do Norte Fluminense (Permit Number: 98).

### 3. Tachyzoites of *Toxoplasma gondii*

Tachyzoites of *T. gondii* (RH strain) were maintained by inoculation into the peritoneal cavity of mice every 2 or 3 days [5]. After this period, a wash of the peritoneal cavity was performed with Hank's solution followed by centrifugation at 100 g for 5 min. The collected supernatant was centrifuged at 1000 g for 10 min. Parasites were resuspended in DMEM and counted in a Neubauer chamber.

### 4. Isolation of PS^+^ and PS^−^ subpopulations

Parasites were collected from infected mice as described above and 2×10^8^ tachyzoites were incubated with annexin V conjugated to magnetic microspheres (Miltenyi Biotec). After 40 min, the cell suspension was added to a magnetic column, retaining only the PS^+^ parasites; PS^−^ parasites were eluted, collected and saved. PS^+^ parasites were removed from the column using standard manufacturer's instructions.

### 5. Flow cytometry analysis of the subpopulations separation method

After magnetic separation, PS^+^, PS^−^ subpopulations of *T. gondii* and the unseparated total population were incubated with annexin-V - alexa 488 and propidium iodide for 1 h [4]. After incubation, parasites were analyzed in a flow cytometer BD Xcalibur. The analyses on histogram were performed in the WinMDI 2.9 program for PC.

### 6. Activation of peritoneal macrophages and interaction with parasites

Interactions were performed with non-activated (resident) and macrophages activated for 24 h right after adherence. Activation was performed with 50 U/ml of murine recombinant interferon-γ (IFN-γ, Sigma) and 100 ng/ml of *Escherichia coli* lipopolysaccharide (0111:B4 LPS, Sigma). The cells were washed and either PS^−^, PS^+^ or the total population of tachyzoites was added with a ratio of 10 parasites to 1 macrophage for 1 and 24 h. In some experiments macrophages were incubated with crescent µM concentrations of dynasore for 1 h before and during the interaction [21].

### 7. Sample preparation for morphological analysis and determination of the index of infection

After methanol fixation, cells were washed, stained with Giemsa (diluted 10× in distilled water), dehydrated in a series of acetone-xylene solution, mounted on Entellan and observed under an optical microscope Axioplan – ZEISS. Images were captured with an MRc5 AxioCam digital camera and processed with the Axiovision program. The percentage of infected cells and the number of intracellular parasites per macrophage were counted and the infection index was obtained by multiplying both numbers [4], [5]. The number of tight-fitting or loose-fitting vacuoles containing *T. gondii* was also counted. Analysis of variance (ANOVA) was used to compare mean values; a *p*<0.05 was considered significant.

### 8. Scanning electron microscopy

Coverslips containing adhered infected macrophages were fixed for 1 h in 4% recently prepared formaldehyde, 2.5% glutaraldehyde in sodium cacodylate buffer 0.1 M, pH 7.4. Cells were dehydrated in acetone. Cells were critical point dried and had their upper part scraped off with adhesive tape, revealing the internal organization of vacuoles [21], [28]. After that, cells were metalized with gold by sputtering (25 nm thick) and observed in a JSM 6490 LQ Jeol scanning electron microscope.

### 9. Analysis of nitric oxide production

The production of NO was indirectly assessed by reading nitrite in the culture supernatants by the Griess reagent. The supernatants were mixed at a ratio of 1∶1 with the Griess reagent (1 volume of 1% Sulfanilamide in 5% phosphoric acid in deionized water with an equal volume of 0.1% N-[1-naphthyl] ethylenediamine in deionized water). After 10 minutes, the mixture was read in an ELISA reader (540 nm) and quantification of NO production was based on a standard curve sodium nitrite in DMEM [29]. The Student *t* test was used to compare mean values, a *p*<0.05 was considered significant.

### 10. *In vivo* infections

C57BL/6 mice were intraperitoneally inoculated with 1×10^4^ of the PS^−^, PS^+^ subpopulation or the total population of tachyzoites. Deaths of mice were recorded daily and a Kaplan Meier survival curve and statistical analysis performed. Spleens and livers of recently dead mice were collected and processed for transmission electron microscopy.

### 11. Light microscopy, transmission electron microscopy and quantification of the cell types in spleen and liver of infected mice

LLC-MK2 and macrophages interacted with the PS^−^ or PS^+^ subpopulations of *T. gondii* for 30 min were washed and fixed with the same solution used for scanning electron microscopy (item 8). Small fragments of recently obtained spleens and livers were fixed with the same solution. Samples were washed, post-fixed with 1% osmium tetroxide, dehydrated with acetone and embebbed in epoxy resin. Semi-thin sections were stained with 1% toluidine blue solution (light microscopy) and ultrathin sections (80 nm) were stained with uranyl acetate and lead citrate (transmission electron microscopy). Ultrathin sections were observed in a Tecnai Spirit transmission electron microscope at 120 KV.

For quantification of the cell types found in spleen and liver of infected mice (with the total tachyzoite population, the PS− or the PS+ subpopulation), alternative semi-thin sections of both tissues were obtained from 2 infected mice that had recently died in the groups. A total of six sections per animal from each group were stained with 1% toluidine blue solution and cells were counted in 4 randomly chosen microscopic fields. Mean numbers of cells were compared between tissues from mice infected with the total population and the PS subpopulations. The Student *t* test was used to compare significant differences (*p*<0.05) of the mean values.
